# XDR typhoid in Pakistan: A threat to global health security and a wake-up call for antimicrobial stewardship

**DOI:** 10.1371/journal.pntd.0013067

**Published:** 2025-05-12

**Authors:** Muhammad Ahmed Abdullah, Babar Tasneem Shaikh, Maryam Ashraf, Shahzad Ali Khan

**Affiliations:** School of Public Health, Health Services Academy, Islamabad, Pakistan; University of Abuja Faculty of Veterinary Medicine, NIGERIA

## Abstract

Extensively drug-resistant (XDR) typhoid, caused by *Salmonella enterica* serotype Typhi, has emerged as a critical global health security threat, with Pakistan, particularly Sindh province, at its epicenter. The misuse of antibiotics, inadequate diagnostic tools, and poor water and sanitation infrastructure have created ideal conditions for the rise of antimicrobial resistance (AMR). XDR typhoid strains resistant to multiple first-line antibiotics have been linked to environmental contamination, with urban areas like Karachi demonstrating high rates of waterborne transmission. International travel has amplified this threat, exporting cases to countries including the United States, the United Kingdom, and Canada, thus highlighting its global implications. This commentary examines the historical context of typhoid treatment, the drivers of AMR in Pakistan, and the critical role of antimicrobial stewardship in combating XDR typhoid. It advocates for an integrated approach that would encompass improvements in water quality, expanded vaccination coverage with typhoid conjugate vaccines (TCVs), and stringent audit of antibiotic prescription practices. Immediate local and global action is needed to contain this public health crisis and prevent the resurgence of typhoid as a largely untreatable disease. This situation underscores the urgency of addressing AMR to safeguard global health security.

## Background

Millions of people worldwide face the growing threat of antimicrobial resistance (AMR), a challenge that is even more severe in developing countries [1]. Typhoid fever, caused by *Salmonella enterica* serotype Typhi, has been a long-standing global health threat, with devastating effects on low- and middle-income countries [2]. The disease has not only resulted into a significant toll of morbidity and mortality but has also contributed to the phenomenon of AMR. Moreover, it has had economic impact, social disruption, surveillance and vaccination challenges, pressure on healthcare system and has posed threat to the global health security. The evolution of typhoid treatment over the past century has emulated the advancements in medical science, evolving from an era where typhoid was often fatal due to the lack of effective treatments, to one where antibiotics and vaccines made it a preventable and treatable condition [3]. However, we are now facing a distressing recession; the emergence of extensively drug-resistant (XDR) typhoid, which threatens to drop us back into a period of vulnerability to this once manageable disease [4]. This specific Typhoid fever is caused by Salmonella Typhi strain which are resistant to all the recommended antibiotics for typhoid fever. Pakistan, particularly the Sindh province, has emerged as the epicenter of a growing global health crisis driven by XDR typhoid, a strain resistant to multiple first-line antibiotics [5]. The widespread misuse of antibiotics, compounded by poor water quality and inadequate sanitation, have driven the rise of AMR [6]. This commentary will explore how we arrived at this perilous situation, the role Pakistan plays as a reservoir for XDR typhoid, and the critical steps that need to be taken locally and globally, to prevent the further spread of this public health threat.

## Historical context of typhoid treatment

A century ago, typhoid fever was a deadly illness with limited treatment options. Before the antibiotic revolution, the management of typhoid relied on supportive treatment, which often failed to thwart mortality. The development of chloramphenicol in the 1940s revolutionized the treatment of typhoid, reducing mortality significantly. This was followed by the introduction of other effective antibiotics, including ampicillin and trimethoprim-sulfamethoxazole. These drugs provided hope and helped turn the tide against a deadly disease [7]. However, by the late 20th century, the misuse of antibiotics began to erode the efficacy of these treatments. Maladministration of antibiotics in the healthcare system, coupled with a lack of robust regulations, led to the rise of multidrug-resistant (MDR) strains of S. Typhi. These strains resisted traditional treatments, forcing reliance on newer, more expensive drugs like fluoroquinolones and cephalosporins. Unfortunately, over the past decade, even these drugs have become ineffective in many parts of the world, particularly in Pakistan, where XDR typhoid strains resistant to fluoroquinolones, third-generation cephalosporins, and azithromycin, have taken hold [8].

## The role of antimicrobial misuse in the rise of XDR typhoid

AMR is often referred to as the silent pandemic [9], and Pakistan exemplifies this issue in the case of XDR typhoid. Decades of indiscriminate and inappropriate antibiotic use in both healthcare and agriculture have accelerated the development of resistant bacteria [10]. In Pakistan, the over-the-counter availability of antibiotics and the non-judicious prescription of these drugs often without proper diagnosis have created fertile ground for resistance [11]. Additionally, the obsolete Typhidot test, which lacks accuracy and specificity, is still widely used in Pakistan despite recommendations for more reliable diagnostic methods. This test frequently produces false positives, leading to unnecessary antibiotic treatments and contributing to AMR [12]. The reliance on such inaccurate diagnostics perpetuates a cycle of over-prescription and drives resistance.

## Environmental and sanitary factors: A breeding ground for typhoid

The XDR typhoid outbreaks in Pakistan are aggravated by poor water quality and sanitation concerns. Access to clean drinking water is inadequate in many parts of the country, especially in urban areas such as Karachi, which faces an acute water scarcity problem. Karachi’s burgeoning population is forced to rely on the illegal water tanker trade, which often supplies contaminated water [13]. Pakistan’s limited water treatment infrastructure and poor waste management systems allow for fecal contamination of water sources, creating an ideal environment for typhoid to spread [14]. Environmental samples from the city have repeatedly tested positive for drug-resistant S. Typhi, further implicating waterborne transmission as a key factor in the spread of both MDR and XDR typhoid [15]. The population’s reliance on untreated water means that waterborne diseases, including typhoid, remain endemic. The spread of XDR strains through environmental contamination poses a major health risk, not only to Pakistan but to neighboring regions and the wider world. XDR strains of diseases like typhoid pose a global health risk due to the growing interconnectedness of the world, particularly in areas where people frequently cross borders for work or trade. XDR typhoid doesn’t respect political boundaries, and its spread can cause outbreaks in regions outside the immediate source area, leading to international health crises.

## Global health security and the threat of international spread

The rise of XDR typhoid in Pakistan is not a local problem, it is a global one. Pakistan has already seen cases of XDR typhoid exported to other countries through international travel. Reports of travelers returning to the United States, the United Kingdom, and Canada with XDR typhoid have highlighted the transnational threat posed by AMR [16]. In an interconnected world, resistant strains do not respect borders, and the failure to control XDR typhoid in Pakistan has the potential to cause international repercussions. If Pakistan fails to control the spread of XDR typhoid, the country could face travel restrictions and economic consequences as other nations seek to protect themselves from imported cases. The issue of XDR typhoid, therefore, is not just a national crisis but a global health security threat.

## Integrated approach, vaccination, and addressing water quality

Addressing the XDR typhoid crisis requires a comprehensive approach that includes improvements in water quality, vaccination, and antibiotic stewardship. One Health approach, which recognizes the interconnectedness of human, animal, and environmental health, is critical to combating the spread of AMR [17]. Improving water, sanitation, and hygiene (WASH) standards must be prioritized to reduce the environmental transmission of typhoid. The government must invest in infrastructure to ensure that all citizens have access to clean drinking water, particularly in urban centers. Moreover, vaccination is a key tool in preventing typhoid infections. Effective antibiotic stewardship is key to preventing further resistance. Health facilities should adopt strict protocols for prescribing antibiotics, ensuring that they are used appropriately, both in terms of dosage and duration, to avoid contributing to the spread of resistance. International collaboration is essential in tracking the global spread of XDR typhoid. Continued investment in research for new control strategies, such as bacteriophages (viruses that target bacteria), probiotics, or novel vaccines, could offer alternative ways to combat drug-resistant strains of S. Typhi [18]*.*

There are certain policy advocacy points to consider. Efforts must be made to expand vaccination campaigns, especially in high-risk areas where outbreaks are common. The introduction of the typhoid conjugate vaccine (TCV) in Pakistan’s routine immunization schedule has been a critical step forward, but coverage remains incomplete [19]. Vaccinating children and vulnerable populations can prevent the spread of XDR typhoid and reduce reliance on antibiotics. Promoting the rational use of antibiotics is essential. Healthcare providers need to be trained in proper prescription practices, and stringent regulations must be enforced to prevent the over-the-counter sale of antibiotics [20]. Diagnostic accuracy should be improved by phasing out the Typhidot test in favor of more reliable methods like blood culture testing, which can guide appropriate treatment and reduce unnecessary antibiotic use. A joint and concerted effort on an international scale is required to restrain the XDR outbreak before it intensifies and leads us back to the pre-antibiotic era [21].

## Conclusion

The emergence of XDR typhoid in Pakistan should be cautioning to the global community about the dangers of AMR. This issue demands urgent attention and coordinated action across sectors. Pakistan policy echelons must address the country’s water quality issues, promote vaccination, and improve diagnostic and antibiotic prescription practices to prevent the further spread of XDR typhoid. The global health community must also recognize the threat posed by this resistant strain and provide support for containment efforts. The fight against typhoid is not over, and without immediate action, we risk entering an era where this disease once again becomes untreatable.
